# Sclerosing Extramedullary Hematopoietic Tumor (SEHT) Mimicking a Malignant Bile Duct Tumor-Case Report and Literature Review

**DOI:** 10.3390/medicina57080824

**Published:** 2021-08-16

**Authors:** Sorin Dema, Fulger Lazar, Robert Barna, Amadeus Dobrescu, Alis Liliana Carmen Dema, Oana Popa, Ioana Ionita, Sorina Maria Taban

**Affiliations:** 1 Radiotherapy Service, Emergency Clinical City Hospital, 300079 Timisoara, Romania; sorindema@yahoo.com; 2 Researching Future Surgery 2 Research Center, University of Medicine and Pharmacy Victor Babes, 300041 Timisoara, Romania; lazarfulger@yahoo.com (F.L.); amadeusdobrescu@yahoo.com (A.D.); 3 Anapatmol Research Center, University of Medicine and Pharmacy Victor Babes, 300041 Timisoara, Romania; barna.robert27@gmail.com (R.B.); taban_oana89@yahoo.com (O.P.); sorinataban@yahoo.com (S.M.T.); 4 Department II Microscopic Morphology–Discipline of Morphopathology, University of Medicine and Pharmacy Victor Babes, 300041 Timisoara, Romania; 5 Multidisciplinary Research Center for Malignant Hematological Diseases, University of Medicine and Pharmacy Victor Babes, 300041 Timisoara, Romania; mdioanaionita@yahoo.com

**Keywords:** sclerosing extramedullary hematopoietic tumor, chronic myeloproliferative disorder, extrahepatic bile ducts, immunohistochemistry

## Abstract

*Introduction*: Sclerosing Extramedullary Hematopoietic Tumor (SEHT) is a very rare lesion associated with chronic myeloproliferative disorders (CMPD). SEHT can mimic morphologically, both macroscopically and microscopically, a wide variety of tumors/lesions. *Case presentation*: We present the case of a female patient diagnosed with gallstones for which surgery was decided. Intraoperatively, a malignant tumor of extrahepatic bile ducts was suspected. A frozen section examination raised the suspicion of a mesenchymal tumor or an inflammatory pseudotumor. The histological evaluation of the permanent sections, supplemented with an immunohistochemical investigation (IHC), was the one that established the diagnosis of SEHT, based on the presence of areas of sclerosis, atypical CD31+ megakaryocytes, myeloid and erythroid elements. *Conclusions*: The authors present the difficulties of a morphological diagnosis on the frozen section and on permanent sections in the absence of relevant clinical information and make a review of the literature data dedicated to the subject.

## 1. Introduction

Sclerosing Extramedullary Hematopoietic Tumor (SEHT), an extremely rare and difficult-to-diagnose lesion, was first described in 1982 [1] and was later reported in case reports [1,2,3,4,5,6,7] or small series of cases [1,8], usually in elderly patients with CMPD, especially primary myelofibrosis (PMF) [5].

We present the case of a female patient in whom the clinical symptoms and the ultrasound examination indicated gallbladder lithiasis, the intraoperative aspects confirming the lithiasis while at the same time raising the suspicion of a malignant extrahepatic bile duct tumor which was excluded during the histopathological examination of the permanent sections that established the diagnosis of SEHT.

## 2. Case Presentation

A female patient, aged 69, with a significant personal pathologic history—appendicectomy, total hysterectomy with bilateral adnexectomy for uterine fibroids, grade-3 essential hypertension, chronic ischemic heart disease, degenerative rheumatism, kidney stones, gallstones detected eight years ago, recurrent depression—was admitted at the Surgery Clinic for pain in the right and left upper quadrant, nausea, vomiting, bitterness in the mouth, headache, sweating, asthenia, lethargy, fatigue, weight loss and exertional dyspnea.

The clinical examination revealed a painful abdomen on palpation in the right hypochondrium and epigastrium and confirmed splenomegaly (6 cm below the costal rim). The abdominal ultrasound showed a hypotonic cudated gallbladder, with large amount of biliary sediment and stones, normal choledochus and splenomegaly with a homogeneous looking spleen. During the imaging techniques, the esophagus, stomach and duodenum showed no pathological changes.

The whole blood analysis determined at admission is presented in Table 1. Anemia and leukocytosis with lymphocytosis and monocytosis were maintained throughout the hospitalization. The rest of the lab tests were in the normal range.

**Figure 1:**
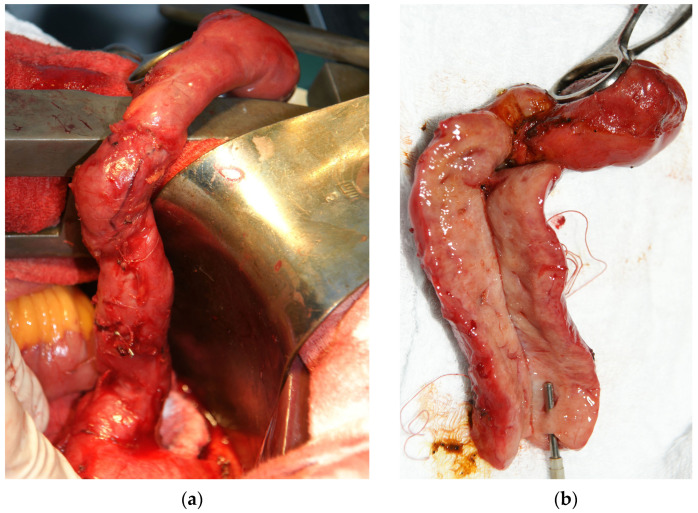
(**a**) Intraoperatively, a long and filiform cystic duct was observed with (**b**) a thick wall noticed on the cut section.

The surgical treatment was decided by using a laparoscopic intervention. The intraoperative exploration detected adhesions that were lysed, a gallbladder with a thickened wall (about 0.5 cm) and a tumor-looking mass involving the cystic duct (Figure 1a), left, right and common hepatic ducts, that could not be dissected laparoscopically, which imposed a conversion to the classic surgical approach. The cystic duct presented a length of approx. 8 cm and a diameter of approx. 1 cm, with a filiform lumen. The common, right and left hepatic ducts also had a thickened wall (about 1 cm) and filiform lumen. At the hepatic hilum, lymph nodes of approx. 1–1.5 cm were detected. A classic anterograde cholecystectomy was performed, and the gallbladder and cystic duct were sent to the pathology department for a frozen section examination. The pathological report described a gallbladder/cystic duct with a benign looking mucosa but with diffuse involvement of the wall by a mesenchymal proliferation with bizarre stromal cells as well as some multinucleated and polymorphic inflammatory cells. The diagnosis was delayed until the evaluation of permanent sections from paraffin-embedded tissue.

Upon a gross examination of the surgical specimen, a 7 cm × 2 cm gallbladder was described, with slightly thickened walls, especially in the fundic region (0.4 cm), containing multiple small black stones and a long cystic duct (10.5 cm), very thickened (1.9 cm), in the shape of a fleshy pink-whitish cord with yellow areas and a reduced lumen (Figure 1b); the lymph nodes in the hepatic hilum had dimensions of 1.7 cm × 0.8 cm × 0.4 cm and a gray color with brown and whitish areas.

The tissue fragments selected for microscopic evaluation were processed according to the usual technique, by paraffin embedding and Hematoxylin & Eosin (HE) staining.

**Figure 2:**
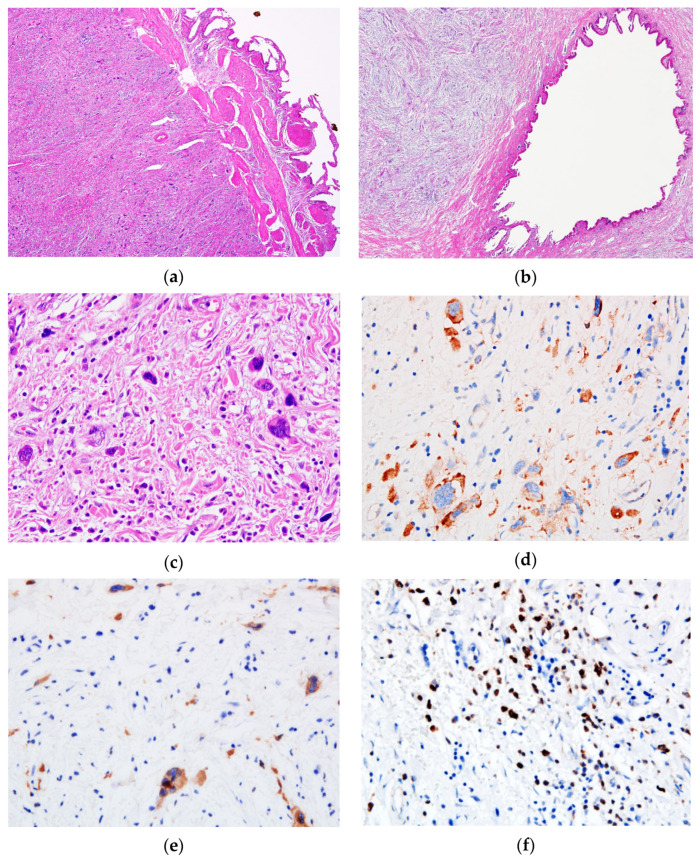
Microscopic features of the tumor. The (**a**) gallbladder and (**b**) cystic duct presented a thickened wall with fibro-myxoid stroma (Hematoxylin & Eosin (HE), ×4). (**c**) Through a high power view, the tumor is composed of isolated or small clusters of atypical megakaryocytes—hyperchromatic nuclei and abnormal chromatin clumping—as well as small cellular elements representing myeloid and erythroid precursors (HE, ×40). Atypical megakaryocytes stained positive for (**d**) CD31 and (**e**) CD61 antibodies, while granulocytic precursors showed a positive expression of (**f**) Myeloperoxidase (×40).

Upon microscopic examination, the mucosa of the gallbladder and the cystic duct showed no significant changes (areas of atrophy and exulceration of the epithelium, discrete chronic inflammatory infiltrate in the chorion); instead, most of the wall’s own structures (partially preserved muscle layer) (Figure 2) were replaced by a mass of fibro-myxoid to sclerotic tissue containing thick collagen strands, with dispersed large cells, isolated or in small clusters, with significant atypia—modified N/C ratio, large, multilobate/multiple, hyperchromatic nuclei, with abnormal chromatin clumping, and small cellular elements representing myeloid and erythroid precursors, lymphocytes, scarce eosinophils and plasma cells. No mitosis, no necrosis and no perineural or lympho-vascular invasion were identified. The sampled lymph node in the hepatic hilum showed a preserved follicular architecture, with the presence of bizarre uni- and multinucleated giant cells in the dilated sinuses.

As the aspects evaluated on the usual stained slides did not allow for the diagnosis to be established, the decision was made to perform an immunohistochemical (IHC) investigation. Additional sections from one selected paraffin block that included the described lesion were cut and placed on super frost ultra plus slides to prevent their detachment during antigen unmasking procedures. Then, the slides were dried for 24 h at room temperature and for 1 h at 56 °C, after which they were placed in a water bath for 20 min at 98 °C and then allowed to cool down. Subsequently, the slides were incubated with the prediluted (ready-to-use—RTU) primary antibodies, which are mentioned below (Table 2), with the exception that the monoclonal mouse anti-myeloperoxidase antibody was diluted at 1:100. The visualization of the reaction was performed with the Universal LSAB2 HRP system (Dako) and Ultravision Polymer HRP system (Novocastra) with Diaminobenzidine (DAB), and the nuclei were counterstained with hematoxylin.

The IHC profile of the atypical cells is also presented in Table 2. Myeloperoxidase (MPO) stained the granulocytic precursors. No CD34 positive blast cells were detected. Numerous small cells positive for CD15 were detected.

The corroboration of morphological aspects evaluated on the usual stained slides with the data provided by the IHC reactions established the diagnosis of fibrous hematopoietic tumor/SEHT most probably in the context of a myelofibrosis/myeloproliferative syndrome, which determined the hospitalization of the patient in the Hematology Department for diagnosis and therapy. At that time, the laboratory investigations showed: ESR—104 mm/h; erythrocytes—2.97 × 10^6^/µL, Hb—7.4 g/dL, Ht—24.6%, MCV—82.8 fL, MCH—24.9 pg, MCHC—30.1 g/dL, platelet count—211 × 10^3^/µL. The peripheral blood smear showed: myelocytes—2%, nonsegmented neutrophils—2%, segmented neutrophils—66%, basophils—4%, lymphocytes—15%, monocytes—10%, blasts—1%, nucleated red blood cells—10/100 leukocytes; the red cell morphology showed frequent dacrocytes, polychromatophilic cells, frequent erythocytes with basophilic stippling, single and multiple Cabot rings; the platelet morphology revealed platelet anisocytosis and macro-megathrombocytes.

The diagnosis of SEHT in the context of the abovementioned hematological changes points to PMF, but unfortunately we do not have data on the result of an osteomedullary biopsy for this patient.

Postoperatively, the patient presented bleeding at the level of the wound, for which blood and plasma were administered with the improvement of the general condition, and she presented also a subhepatic abscess for which surgery was performed. The patient died four months later.

## 3. Discussion

Chronic myeloproliferative disorders (CMPDs) are clonal myeloid stem cell disorders characterized by the proliferation of one or more myeloid cell lines [8] and include chronic myeloid leukemia (CML), polycythemia vera (PV), essential thrombocythemia (ET) and primary myelofibrosis (PMF) [9].

Primary myelofibrosis (PMF), a myeloproliferative neoplasm characterized by the abnormal proliferation of megakaryocytes and granulocytes in the bone marrow, consists, in the established form of the disease, of bone marrow fibrosis and foci of extramedullary hematopoiesis (EMH) [10].

Extramedullary hematopoiesis, defined as the presence and growth of immature hematopoietic cells in other locations than the bone marrow in some benign or malignant diseases, is a consequence of a low production of hematopoietic cells in the bone marrow from various causes [3,11]. Extramedullary hematopoiesis is common in patients with CMPD, especially PMF [4], and more commonly affects the liver, spleen and lymph nodes, with hepatosplenomegaly, sometimes significant, and lymphadenopathy; however, it has been reported in other rare locations: heart, thymus, lung, pleura, dura mater, paraspinal region, gastrointestinal tract, peritoneum, mesentery, retroperitoneum, kidneys, adrenal glands, pelvis, uterus, breast, skin and soft tissue [2,4,5,7,8,9,12,13,14,15,16].

Unlike EMH, from which it should be distinguished, the fibrous hematopoietic tumor described under this name by Beckman and Oehrle in 1982 [1] and later called SEHT [8] forms a solid tissue mass by reactive fibroblast proliferation, it is less cellular and has the peculiarity of exhibiting the presence of atypical megakaryocytes [4,13].

EHT is a difficult lesion to diagnose in the absence of information regarding the predisposing hematological conditions, on the one hand due to the rarity of the lesion (and thus the reduced familiarization of the pathologist with the lesion) and on the other hand due to the particular microscopic appearance.

The lesion has been reported in various locations: digestive tract with the involvement of the liver, ligamentum teres, spleen, lesser omentum and mesentery, retroperitoneum, kidney, adrenal gland, pelvis, skin, lacrimal gland, orbit, thyroid gland, lung, breast, heart and lymph node [1,2,3,5,6,7,8,13,17,18].

The sclerosing extramedullary hematopoietic tumor appears as a single or multiple tumor (pseudotumor) lesion of various sizes going up to 15/6 cm, with a rubbery consistency, pinkish or white or yellow-tan color, circumscribed and sometimes multilobulated, which can adhere to and/or infiltrate the surrounding tissue [3,5].

In all reported cases, the lesion was not clinically suspected, the correct diagnosis representing the prerogative of the histopathological examination including the IHC investigation.

Histologically, SEHT consists of aggregates of polymorphic cells—immature and mature elements of the three hematopoietic series with atypical megakaryocytes that have large, multilobate/multiple, hyperchromatic, ink-blot-like nuclei and occasionally an eosinophilic cytoplasm. This cellular component is dispersed in a fibro-myxoid to sclerotic stroma, sometimes with entrapped adipose tissue [13]. Atypical megakaryocytes are IHC positive for factor VIII, CD31, CD61 and CD41 [5]. Granulocytes are highlighted by the positive reaction for myeloperoxidase, and erythrocyte precursors by the positivity for glycophorin A [4] and hemoglobin [8].

In view of our knowledge, the case presented is the only one in the Romanian and English language medical literature in which the lesion involving extrahepatic bile ducts grossly mimics a malignant bile duct tumor.

Due to the presence of fibrosis and atypical cells, the spectrum of differential diagnoses is quite wide and includes: EMH foci that are more cellular, with reduced fibrosis and without atypical megakaryocytes; adrenal myelolipoma, which combines hematopoietic elements with abundant mature fat tissue but not with sclerosis, and which is devoid of atypical megakaryocytes; an inflammatory myofibroblastic tumor can be ruled out based on the absence of hematopoietic elements and on the positive reaction for ALK; GIST can be excluded due to the lack of reactivity for CD117 in atypical cells from SEHT; sarcomas such as leiomyosarcoma, malignant peripheral nerve sheet tumor, myxofibrosarcoma, myxoid or sclerosing liposarcoma can be excluded based on the presence of mitosis and necrosis and the presence of lipoblasts in liposarcoma; myeloid sarcoma is characterized by a high cellularity with a predominance of blasts, the absence of sclerosis and a positive reaction of the tumor cells for lysosim, CD43, CD68; Hodgkin’s lymphoma with lymphocyte depletion, an important imitator, can be excluded based on the presence of other hematopoietic elements and on the negative reaction for CD30 and CD15 in atypical cells from SEHT; sarcomatoid carcinoma, unlike SEHT, is devoid of granulocyte and erythroid precursors and demonstrates a positive reaction for CK in the atypical cells [3,8,11,12].

The prognosis of patients with SEHT is variable [3] and is generally related to the underlying disease, sometimes with long periods of stable disease that do not justify aggressive local therapy [2], other times with a short survival possibly related to advanced disease [8]. In fact, the lesion itself could be considered a marker of advanced disease [8,13,16]. Among the unfavorable prognostic factors, the following are mentioned: advanced age, anemia, leukocytosis, thrombocytopenia [19] and skin involvement [16]. The presence of an abnormal karyotype seems to be an independent factor of unfavorable prognosis, correlated with acute conversion [20].

The aspects reported in this study have to be seen in light of some limitations because we did not have access to the results of a bone marrow biopsy and molecular tests could not be performed. Despite these limitations, which are more useful for the management of the underlying disease, the case we reported is about SEHT involving the gallbladder, with an emphasis on the difficulties encountered by surgeons and pathologists in establishing the correct diagnosis of this rare tumor.

## 4. Conclusions

The present case contributes to the expansion of current knowledge about SEHT, an extremely rare lesion that can affect the extrahepatic bile ducts. Morphological diagnosis is difficult and requires familiarization of the pathologist with this entity, careful examination of the lesion cells and the use of an appropriate panel of antibodies, thus avoiding the misinterpretation of megakaryocyte atypia and, implicitly, a false diagnosis. Establishing the diagnosis of SEHT in a patient without a history of hematological disorder requires careful investigation to detect the underlying disease.

## Figures and Tables

**Table 1:** Blood analysis determined at admission.

Blood Parameter	Value	Laboratory Range
WBC ^1,2^	28 × 10^3^/µL ^3^ → 31.3 × 10^3^/µL → 20.9 × 10^3^/µL → 16.6 × 10^3^/µL → 14.1 × 10^3^/µL → 11.3 × 10^3^/µL	4–9 × 10^3^/µL
Lymphocytes	14 × 10^3^/µL	1–3.2 × 10^3^/µL
Monocytes	4.7 × 10^3^/µL	0.2–0.7 × 10^3^/µL
Granulocytes	9.4 × 10^3^/µL	2.1–6.7 × 10^3^/µL
Erythrocytes	3.64 × 10^6^/µL	4–5.50 × 10^6^/µL
Hemoglobin	9.1 g/dL ^4^	11.5–15 g/dL
Hematocrit	28.5%	35–45%
MCV ^7^	78.3 Fl ^5^	80–100 fL
MHC ^8^	25 pg ^6^	27–32 pg
MCHC ^9^	31.9 g/dL	32–36 g/dL
Platelet count	273 × 10^3^/µL	150–400 × 10^3^/µL
AST ^10^	40 U/L	5–34 U/L

^1^ Evolution of the ^2^ white blood count throughout the hospitalization is presented in the table with a black arrow; ^3^ microliter; ^4^ grams per deciliter; ^5^ femtoliter; ^6^ picogram; ^7^ mean corpuscular volume; ^8^ mean hemoglobin content; ^9^ mean corpuscular hemoglobin concentration; ^10^ Aspartat-Aminotransferase.

**Table 2:** Antibodies used to establish the IHC (immunohistochemical) profile of the tumor and their expression.

Primary Antibody	IHC Expression	Clone	Company
CD31	Positive atypical megakaryocytes	CD31-607	Novocastra
CD61	Positive atypical megakaryocytes	2F2	Novocastra
MPO ^1^	Positive granulocytic precursors; Negative atypical megakaryocytes;	59A5 (1:100, pH6)	Novocastra
PanCK ^2^	Negative	AE1/AE3	Dako/Agilent Technologies
EMA ^3^	Negative	E29	Dako/Agilent Technologies
SMA ^4^	Negative	1A4	Dako/Agilent Technologies
Desmin	Negative	D33	Dako/Agilent Technologies
Vimentin	Negative	V9	Dako/Agilent Technologies
ALK ^5^	Negative	ALK1	Dako/Agilent Technologies
S100	Negative	Polyclonal	Novocastra
CD3	Negative	Polyclonal	Cell Marque
CD10	Negative	56C6	Dako/Agilent Technologies
CD15	Negative	Carb-3	Dako/Agilent Technologies
CD20	Negative	L26	Dako/Agilent Technologies
CD30	Negative	Ber-H2	Dako/Agilent Technologies
CD34	Negative	QBEnd10	Novocastra
CD68	Negative	PG-M1	Dako/Agilent Technologies
CD117	Negative	EP110	Master Diagnostica
Ki-67	<3%	MIB 1	Dako/Agilent Technologies

^1^ Myeloperoxidase, ^2^ Pan Cytokeratin, ^3^ Epithelial Membrane Antigen, ^4^ Smooth Muscle Actin, ^5^ Anaplastic Lymphoma Kinase.

## Data Availability

All data are available on request to the corresponding author.
